# Factors Associated With Age-related Hearing Impairment: A Retrospective Cohort Study

**DOI:** 10.1097/MD.0000000000001846

**Published:** 2015-10-30

**Authors:** Il Joon Moon, Hayoung Byun, Sook-young Woo, Geum-Youn Gwak, Sung Hwa Hong, Won-Ho Chung, Yang-Sun Cho

**Affiliations:** From the Department of Otorhinolaryngology-Head and Neck Surgery, Samsung Medical Center, Sungkyunkwan University School of Medicine (IJM, HB, SHH, W-HC, Y-SC); Biostatistics Team, Samsung Biomedical Research Institute (S-yW); and Department of Medicine, Samsung Medical Center, Sungkyunkwan University School of Medicine, Seoul, Korea (G-YG).

## Abstract

Age-related hearing impairment (ARHI) is a complex degenerative disease in the elderly. As multiple factors interact during the development of ARHI, it is important to elucidate the major influencing factors to understand and prevent ARHI. We aimed to identify risk factors associated with the development of ARHI with a retrospective cohort from 2001 to 2010. The records of the adult subjects over 40 years of age who consecutively underwent a comprehensive health checkup including pure-tone audiometry at the Health Promotion Center were reviewed. During this period, 1560 subjects who underwent pure-tone audiometry more than twice, had no other otologic diseases, and were followed-up more than 2 years were included. A pure-tone average (PTA: 0.5, 1, 2, 4 kHz) was calculated. Development of ARHI was defined as a PTA at follow-up more than 10 dB greater than the baseline PTA. Times to the first development of ARHI were investigated. Overall, 12.7% of subjects developed ARHI within the first 4 years. High blood ionized calcium (hazard ratio [HR] 0.084), albumin (HR 0.239), systolic blood pressure (HR 0.577), thyroid hormone (T3) (HR 0.593), and alpha fetoprotein levels (HR 0.883) were associated with decreased hazard for the development of ARHI. In contrast, high blood high-density lipoprotein (HR 2.105), uric acid (HR 1.684), total protein (HR 1.423), and total bilirubin levels (HR 1.220) were potential risk factors for the development of ARHI. Development of ARHI is common among the aged population, and a variety of factors may interact during this process. The results of this study can be used for counseling of adults at high-risk of developing ARHI with regard to regular audiological check-up.

## INTRODUCTION

Age-related hearing impairment (ARHI), or presbycusis, is a complex and progressive degenerative disease, affecting cognitive, emotional, and social functions in the elderly.^1^ Hearing acuity significantly declines with age, and the prevalence of hearing loss approximately doubles every decade of life from the 2nd through the 7th decade. Using a cut-off of 25 dB HL for the definition of hearing loss, the prevalence of ARHI in aged subjects was 68.1% (>70 years) in a recent epidemiologic study.^2^ In addition, as societies become older, an increasing proportion of population will develop ARHI in the near future.

ARHI does not occur uniformly in all individuals. Although numerous studies have been conducted for decades, there remains no way to predict who is at highest risk, with no definitive strategy for prevention and treatment. Although the exact pathophysiological mechanism of ARHI is still unclear, multiple intrinsic (genetic) and extrinsic (environmental) factors have been proposed to be involved overtime in the development of ARHI.^3^ As multiple factors interact to produce additive effects, it is important to elucidate major influencing factors relevant to ARHI to understand and prevent the progression of ARHI.

A number of cross-sectional studies have provided insight into potential risk factors associated with ARHI. These include male sex, increasing age, hypertension, cardiovascular disease, cerebrovascular disease, smoking, diabetes, and noise exposure.^4–6^ However, these risk factors apparent from cross-sectional studies only provide information of the momentary causality of the condition, and there is no definitive information for a temporal relationship between exposure and outcome. These limitations of cross-sectional studies can be avoided by a longitudinal design.^7^ Few epidemiologic studies have documented the risk factors associated with ARHI longitudinally in adults. Furthermore, no studies have involved comprehensive laboratory profiling and analysis of risk factors in a large population cohort.

In this study, the authors accessed longitudinal data-set from individuals who underwent routine health-checkups including demographic, social, and complete laboratory profiles as well as more than 2 audiometric tests. The aim of this study was to identify risk factors associated with development of ARHI in adults.

## METHODS

### Study Sample

The study subjects were recruited among those aged 40 years or older who underwent a health checkup including audiometry at the Health Promotion Center more than twice between January 2001 and April 2010. A total of 4849 subjects were initially assessed for their eligibility, and 2471 subjects were first excluded due to the short follow-up period (<2 years). Thorough reviews of medical charts were performed in the remaining 2378 subjects and 88 subjects were additionally excluded from the study, because they had a variety of otologic diseases, such as otitis media with effusion or perforated tympanic membrane, which can affect hearing. Thus, 2290 subjects met the inclusion criteria that included no otologic diseases and more than 2 years of follow-up. However, 760 of 2290 subjects who had some missing data were also excluded from the analysis. Finally, 1560 subjects remained for analysis. The use of human subjects and the experimental protocols were reviewed and approved by the Samsung Medical Center Institutional Review Board (2010-09-002-002).

### Collection and Classification of Covariates

Demographics were obtained at the first visit. Personal medical history and lifestyle factors, including cigarette smoking and alcohol consumption, were also determined using a structured questionnaire. Past medical history of cardiovascular diseases, diabetes mellitus, and cerebrovascular diseases were also obtained. Height, weight, diastolic, and systolic blood pressures (SBPs) were measured in all subjects. Body mass index was calculated as weight (in kilograms) divided by height (in meters) squared. In addition, complete laboratory profiles including complete blood count, liver panel, renal panel, lipid panel, tumor markers, serology, ABO blood typing, C-reactive protein, fibrinogen, prothrombin, partial thromboplastin times, and urinalysis were obtained. All blood samples were obtained during the morning after an overnight fast of more than 8 hours.

### Hearing Evaluation

The hearing examination at each visit included pure-tone air-conduction audiometry in sound-treated test booth. All audiometric testing was performed using a GSI-61 Clinical Audiometer (Grason-Stadler, Littleton, MA) and Telephonics TDH-50P headphones (Teledyne Avionics, Charlottesville, VA). The audiometers were calibrated every 6 months in accordance with appropriate American National Standards Institute standards (ANSI, 1996, 2004). Pure-tone air-conduction thresholds were obtained for each ear at 250, 500, 1000, 2000, 4000, and 8000 Hz. If air-conduction thresholds at any frequency exceeded more than 25 decibels (dB), bone-conduction thresholds were obtained and the subject was referred to an otolaryngology outpatient clinic for further evaluation. In the clinic, thorough evaluation including otoendoscopic examination of the tympanic membrane was performed for the referred subject. A pure-tone average (PTA: 0.5, 1, 2, 4 kHz) was calculated for each subject. The outcome of interest was the time to the first ARHI development, which was defined as a PTA at follow-up 10 dB greater than the baseline PTA in either ear.

### Statistical Analyses

Quantitative non-normally distributed variables were expressed as median and the 25th to 75th quartile range. Categorical variables were presented as frequency and percentages. Due to interval censored data, the ARHI development free rates were calculated with the use of the self-consistency algorithm of Turnbull.^8^ To examine the risk of various covariates, including laboratory profiles, on the development of ARHI, univariable and multivariate analyses were performed. We checked a plot of log(−log S(t)) versus time for evaluating Weibull distribution. The plots showed the linearity. Parametric survival model with Weibull distribution for interval-censored data was used to examine the effects of various factors, including laboratory profiles in the development of the first ARHI. Biologically and medically plausible covariates as well as the variables found to be possibly associated (*P* < 0.10) in the univariable analysis were entered in the multivariable analysis model. The continuous variables, such as SBP, low-density lipoprotein, high-density lipoprotein (HDL), blood urea nitrogen, uric acid, thyroid hormone, and alpha fetoprotein were transformed using natural log due to the skewed distribution. Multicollinearity was checked using variance inflation factor. There were no variables with variance inflation factor >4. The variables with Spearman correlation coefficient >0.7 were not included in the analysis due to the multicollinearity problem. *P* values and 95% confidence interval (CI) were corrected by Bonferroni's method due to multiple testing. In all tests, *P* < 0.05 was considered statistically significant. Statistical analysis was performed using SAS 9.3 (SAS Institute, Cary, NC).

## RESULTS

### Demographic Data and Hearing Changes

**FIGURE 1:**
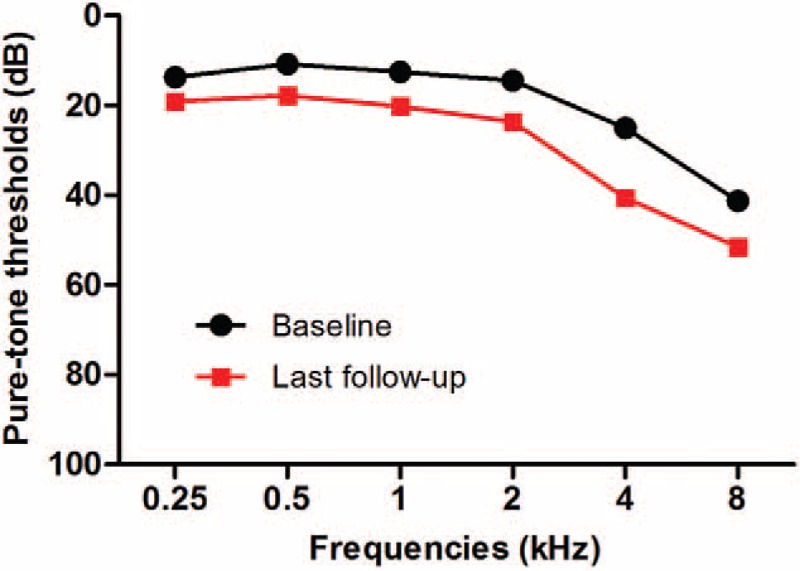
Mean hearing thresholds of each frequency at baseline and last follow-up examinations. The mean pure-tone average (0.5, 1, 2, 4 kHz) changed from 15.8 ( ± 6.4) dB to 25.7 ( ± 10.3) dB between the baseline and last follow-up examinations.

**FIGURE 2:**
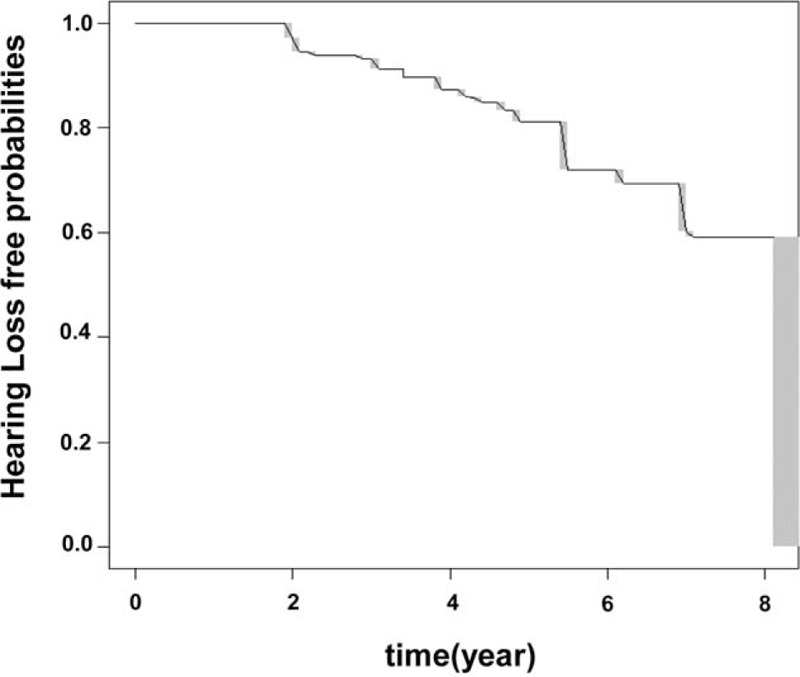
Age-related hearing impairment (ARHI)-development free curve for interval censored data. ARHI-development free curve was plotted for all the subjects using a previously describe nonparametric procedure.^9^ Gray rectangles show the 95% confidence intervals within which the curve would maximize the likelihood.

**FIGURE 3:**
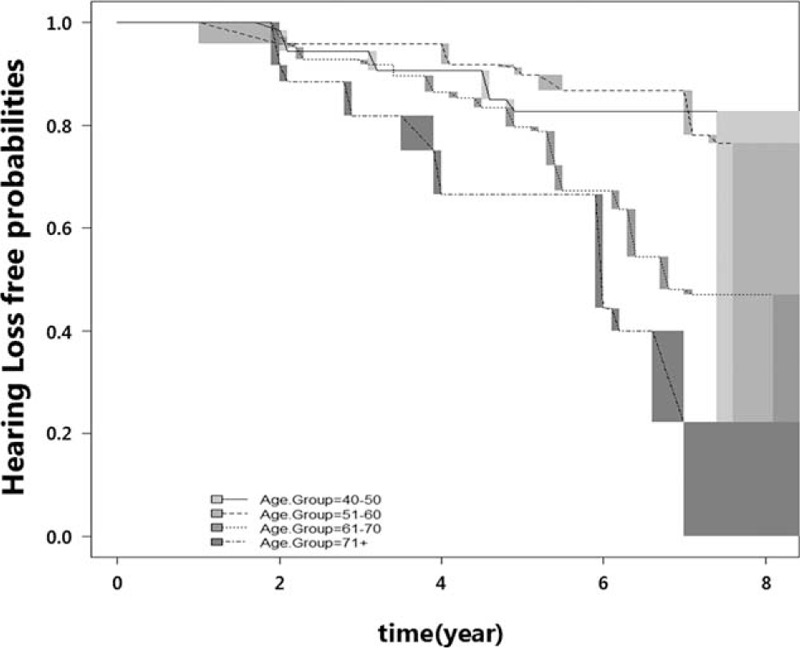
Age-related hearing impairment (ARHI)-development free curve among different age groups. ARHI-development free curve (ie, the cumulative proportion of subjects without hearing impairment) was plotted for all different age groups using the nonparametric procedure.^9^ Gray rectangles show the 95% confidence intervals within which the curve would maximize the likelihood.

From January 2001 to April 2010, 2290 subjects (1673 males, 617 females) were initially screened, and 1560 subjects (1172 males, 388 females) were finally enrolled. The various covariates including laboratory profiles were not significantly different between the population of 2290 subjects that met the inclusion criteria initially and the population of 1560 subjects that were finally included in our study. The median age of subjects at baseline was 65 years (range 50–87 years, interquartile range 50–66 years). The median duration between the first visit and last visit is 4.00 years (range 2.00–8.07 years, interquartile range 2.97–5.17 years). Categorical variables were described by frequencies and percentage and descriptive statistics for continuous variables were described by medians and interquartile ranges in Table 1. Figure 1 shows the mean pure-tone threshold of each frequency in 1560 participants at the baseline and last follow-up examinations. The mean PTA changed from 15.8 (±6.4) dB to 25.7 (±10.3) dB between the baseline and last follow-up examinations (*P* < 0.001). Overall, 12.7% of subjects developed ARHI within the first 4 years, and the probabilities of ARHI development steadily increased up to 41% at 8 years. Figure 2 shows ARHI development-free curve. In order to figure out the difference in ARHI development among different age groups, ARHI development-free curve of different age groups are plotted in Figure 3.

**TABLE 1:**
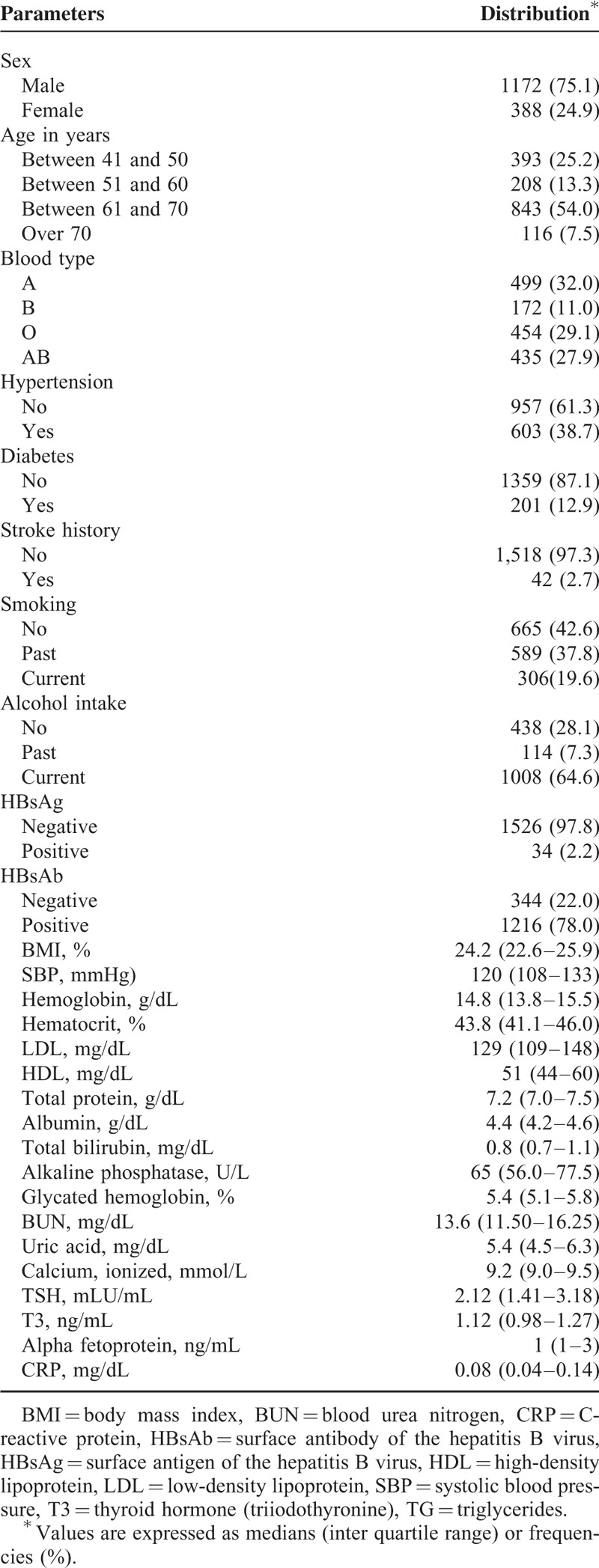
Clinical Characteristics and Laboratory Findings of 1560 Subjects Enrolled in the Study

### Univariable Analysis

Table 2 displays the results of the univariable analysis. Parametric regression models with Weibull distribution revealed that SBP (hazard ratio [HR], 0.544; 95% CI, 0.382–0.775), blood low-density lipoprotein level (HR, 0.777; 95% CI, 0.630–0.958), protein level (HR, 0.804; 95% CI, 0.708–0.912), albumin (HR, 0.298; 95% CI, 0.245–0.363), calcium level (HR, 0.627; 95% CI, 0.578–0.681), thyroid hormone (T3) level (HR, 0.479; 95% CI, 0.373–0.615), and alpha fetoprotein level (HR, 0.866; 95% CI, 0.787–0.952) were significant protective factors of the development of ARHI. In contrast, blood HDL level (HR, 1.441; 95% CI, 1.158–1.793), total bilirubin level (HR, 1.146; 95% CI, 1.011–1.298), blood urea nitrogen level (HR, 1.259; 95% CI, 1.039–1.525), and uric acid level (HR, 1.468; 95% CI, 1.200–1.796) were important risk factors for the development of ARHI.

**TABLE 2:**
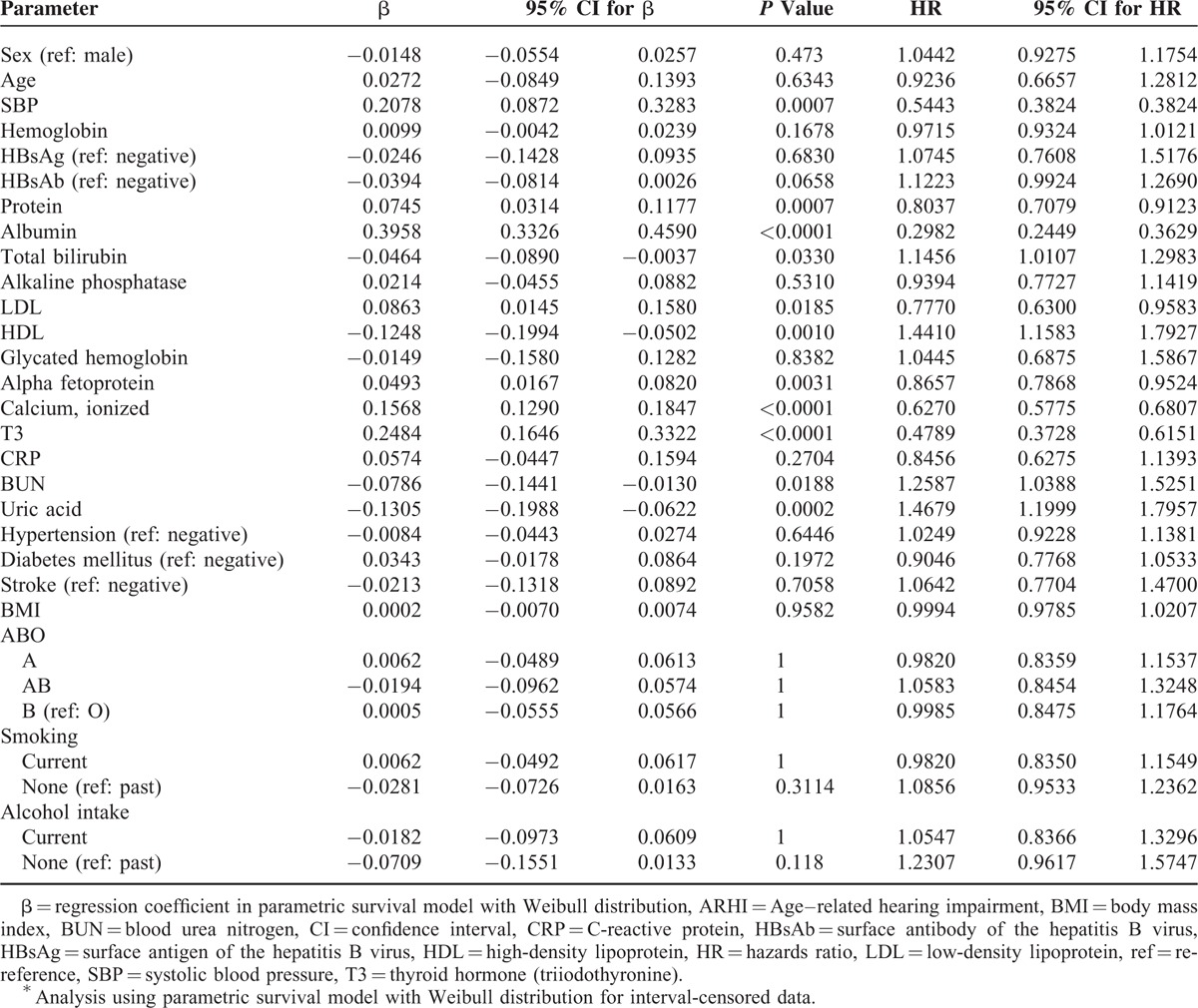
Unadjusted Effects of Each Variable on Development of ARHI^∗^

### Multivariable Analysis

Results of the multivariable parametric regression models with Weibull distribution are reported in Table 3. Of the 12 variables entered into the multivariable model, high blood calcium (HR, 0.084; 95% CI, 0.038–0.186), albumin (HR,0.239; 95% CI, 0.187–0.305), SBP (HR, 0.577; 95% CI, 0.403–0.825), thyroid hormone (T3) (HR, 0.593; 95% CI, 0.458–0.769), and alpha fetoprotein levels (HR, 0.883; 95% CI, 0.803–0.971) were potent protective factors for the development of ARHI. In contrast, high blood HDL (HR, 2.105; 95% CI, 1.673–2.647), uric acid (HR, 1.684; 95% CI, 1.361–2.083), total protein (HR, 1.423; 95% CI, 1.233–1.643), and total bilirubin levels (HR, 1.220; 95% CI, 1.073–1.386) were significantly associated with increased risk for the development of ARHI.

**TABLE 3:**
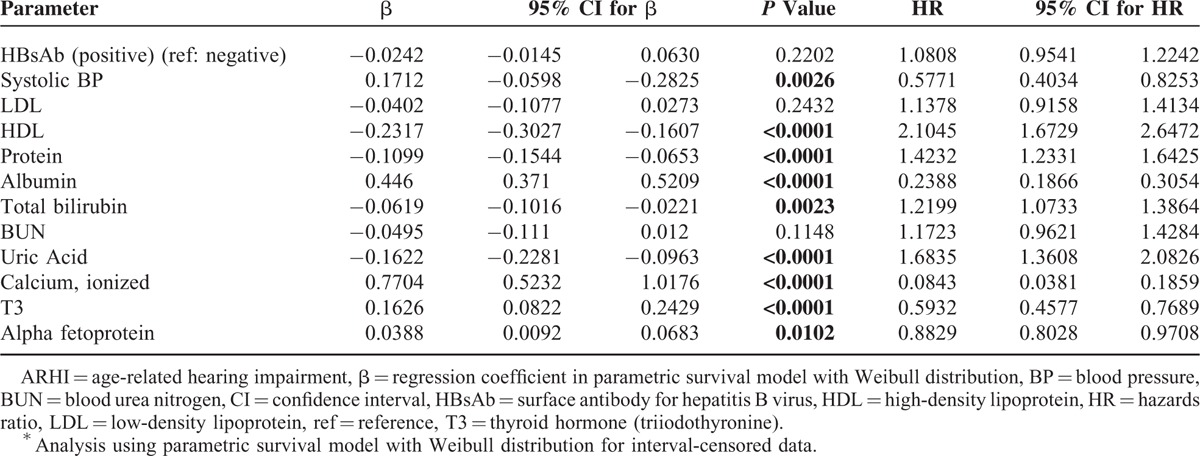
Significant Parameters in Development of ARHI^∗^

## DISCUSSION

The prevalence and severity of ARHI is increasing with age. Besides its high incidence and prevalence, ARHI affects quality of life enormously by reducing communicative relationships, as well as reducing social and emotional interactions.^10^ It has seemed impossible to prevent the development of ARHI, even though potential pathogenic mechanism and risk factors have been uncovered. Revealing potential risk factors could be the first step for the prevention of ARHI, and this study attempted to identify these factors using a large retrospective cohort.

In this study, 44% of adult population over 40 years of age developed 10-dB hearing deterioration within the first 8 years, after the baseline testing. Ten dB corresponds to a 10-fold increase in sound intensity or 3.16-fold increase in sound pressure. Presence of 10 dB HL deterioration in hearing thresholds in both ears of a subject may have an adverse effect on auditory perception in daily conversations. However, there is 5 to 10 dB HL variability in the audiometric threshold testings. This is related to the fact that since threshold estimations are carried out in 5 dB HL steps, 10 dB HL deterioration may not considered as a significant in terms of clinical studies. A previous longitudinal study reported that 5-year progression of hearing loss, defined as 5-dB hearing deterioration, was 51.1% for men and 56.3% for women.^11^ As the definition of hearing change is different (10 vs 5 dB in PTA) and these progression rates were obtained from individuals who have hearing loss (greater than 25 dB HL in either ear) at baseline, no direct comparison is possible. Nonetheless, our data support the reality that adults should expect their hearing to deteriorate with age.

Of note, most of the influential results in this study are novel protective and risk factors for the development of ARHI. Although it is difficult to elucidate the role of key risk factors due to the multifactorial nature and interaction of the different mechanisms, blood calcium, albumin, alpha fetoprotein, and thyroid hormone levels as well as SBP are potent protective factors from multivariable analysis. In contrast, blood uric acid, HDL, protein, and total bilirubin levels are potential risk factors.

In our study, high SBP has protective effect in developing ARHI. However, the median SBP in the population included in this study was 120 (Q1, 108; Q3, 133) mmHg. Thus, high SBP in our study did not correspond to real hypertension and cannot be interpreted as indicating that subjects with hypertension having a decreased risk for the development of ARHI. Furthermore, a previous study reported that arterial hypotension, expressed by low SBP, is significantly related with poor high-frequency hearing in the elderly.^12^ Blood pressure-dependent sympathetic effect on the labyrinthine circulation might be responsible for this association.^13^

Human blood albumin is crucial for heme-iron scavenging, providing antioxidant effect against free heme-iron oxidative damage. A previous study has shown that albumin is responsible for more than 70% of total blood antioxidant properties.^14^ Oxidative stress plays an important role in the development of ARHI.^3^ Current evidence supports the view that the cumulative effect of oxidative stress could induce damage to mitochondrial DNA in the cochlea and that the decrease in mitochondrial function can result in apoptosis of the cochlear cells. Therefore, it is postulated that the strong antioxidant properties of blood albumin might contribute to protect cochlear damage against accumulated oxidative stress overtime.

Thyroid hormones (THs) are synthesized by the thyroid gland and have been classified into 2 major forms: T_3_ (3,5,30-triiodol-thyronine) and T_4_ (3,5,30,50-tetraiodo-l-thyronine). T_3_ is the active form of THs, as T_3_ binds to thyroid hormone receptors (TRs) with a greater affinity than T_4_. THs play significant roles in fetal development of nervous system and hearing as well as the maintenance of brain function and regulation of neuopsychological function in adults. Especially, TH is a critical regulator of the cochlear motor protein prestin.^9^ Previous epidemiologic study in school-age population also supported the relationship between the elevated auditory thresholds and decreased thyroid function.^15^ The protective effect of T_3_ on ARHI may be attributable to this strong relationship between thyroid function and the development and maintenance of hearing.

Hair cells and spiral ganglion neurons (SGNs) have several types of calcium channels, such as L- and T-type voltage-gated calcium channels.^16^ These calcium channels and signaling pathway is associated with the age-related loss of SGNs, and calcium dysregulation is related to neuronal death during aging.^17^ It has also been reported that blood calcium level was significantly lower in patients with presbycusis than in normal subjects.^18^ Thus, demineralization of the petrous temporal bone associated with bone mass loss with aging has been suggested as one biologic factor for ARHI.^19^ High blood calcium level may prevent bone demineralization and subsequent hearing impairment overtime.

Human AFP has an antiinflammatory effect and immunomodulating effect. Especially, AFP inhibits local central nervous system inflammation by downregulating the expression of major histocompatibility complex class II molecules, which are key elements in antigen presentation, and the chemokine receptor-5, which is crucial for leukocyte migration to inflammatory sites. Furthermore, AFP suppresses the production of myelin-specific antibodies and IgG, and shows a beneficial effect on central nervous system demyelination and axonal damage.^20^ The protective effect of AFP on ARHI may be related with these strong antiinflammatory and immunomodulating properties.

In contrast, uric acid may induce neurotoxicity through different mechanisms. An elevated uric acid level is a risk factor for vascular disease and hypertension.^21^ The vascular compromise associated with elevated uric acid level could be a potential risk factor for ARHI. The cochlea has a vascular supply without collateral vessels and can be highly sensitive to minimal blood flow reduction.^22^ A previous study revealed significant abnormalities in otoacoustic emissions (OAEs) observed at higher frequencies (4 and 5 kHz) in patients with normal hearing (PTA < 20 dB), suggesting an association of subclinical cochlear dysfunction (early cochlea damage especially in outer hair cells) with hyperuricemia.^23^

Bilirubin may induce neurotoxicity through different mechanisms, such as excitotoxicity or activation of nitric oxide synthesis.^24^ A recent study involving guinea pigs demonstrated that bilirubin can cross the blood–brain barrier and can damage the peripheral auditory system including SGNs and inner hair cells.^25^ Therefore, highly maintained blood bilirubin level may directly induce cochlear damage and subsequent development of ARHI.

HDL has an antiatherogenic effect. In an extensive review of the literature, Ray^26^ questioned the association between hyperlipoproteinemia and ARHI. Recently, neurodegenerative effects of HDL have been reported. HDL shows antineuritogenic effects in neuronal cell culture and inhibits the biosynthesis of gangliotetraosyl gangliosides. Lipid peroxidation can cause the formation of HDL macroaggregates and subsequent conformational changes in the protein moieties. These modified HDLs might be able to induce degeneration of neuronal cells.^27^

Plasma viscosity is directly correlated with blood globulin (total protein minus albumin), so high blood total protein levels can lead to high plasma viscosity. Plasma viscosity in patients with sudden deafness is significantly higher than that in control group with normal hearing with a significant correlation between average pure-tone thresholds and plasma viscosity.^28^ Elevated plasma viscosity may decrease cochlear blood flow and thus initiate hearing loss. This is consistent with the finding that lowering plasma viscosity in patients with sudden sensorineural hearing loss is helpful for improving treatment outcome.^29^

One of possible limitations of this study is that the quantification of previous noise exposure was impossible in this study population. Noise induces cochlear damage by reducing blood flow and inducing formation of free radicals.^30^ However, there have been no reports that subjects with previous noise exposure history are prone to ARHI development at this point. In addition, the population in this study was male-dominant and 54% of the population were in their 60s. This is not surprizing, given that this study population was from a health promotion center. Male gender and old age are risk factors for the development of ARHI, so elderly males would be the most vulnerable population for worsening of hearing overtime. The aim of this study was to assess the risk factors of ARHI, and it fits well for the purpose that only participants more than 40 years of age were included.

Overall, the key attributes of this study lie in the large cohort, providing detailed and standardized audiometric data over several years, as well as in the comprehensive range of demographic, clinical, and laboratory variables measured at baseline enabling a thorough evaluation of predictors for the development of ARHI. In a clinical setting, the predictors revealed in this study can be used for counseling patients who have concerns about their further hearing deterioration overtime. Also, it would be reasonable to recommend regular audiological check-up for patients with risk factors for the development of ARHI. Besides clinical aspects, the results in our study could provide further insight into the mechanistic pathways underlying ARHI. Further biological investigation may prove informative in identifying protective factors in ARHI useful in the search for prevention strategies.

## CONCLUSIONS

Our findings suggest that development of ARHI is common in adult population. Patients with high blood calcium, albumin, thyroid hormone (T3), and AFP levels, as well as high SBP may less likely experience worsening of hearing overtime. The prognosis is less favorable for those who have high blood bilirubin, total protein, HDL, and uric acid levels.
